# Supplementary figures and images for: Association of microtubule destabilization with platelet yields in terminally differentiating hiPSC-derived megakaryocyte lines
Source: PLoS One. 2025 Jun 25;20(6):e0326165. doi: 10.1371/journal.pone.0326165 (PMC12194186; doi:10.1371/journal.pone.0326165)

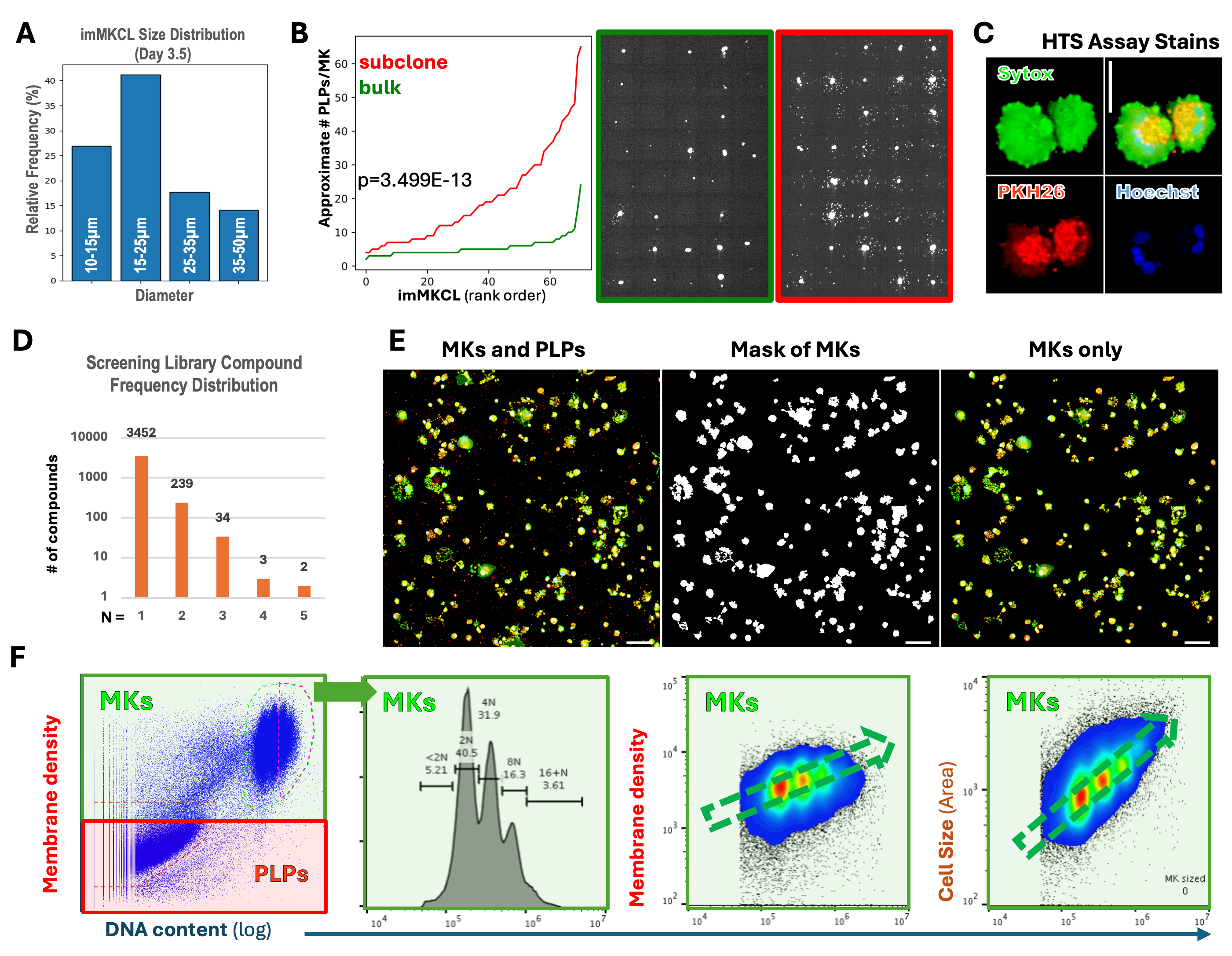

Supplement: S1 Fig — (A) Histogram of the size distribution of imMKCLs (related to Fig 1B; diameters estimated from 20x confocal timecourse imaging data with membrane-dye stained imMKCLs). (B) Rank-order plot comparing platelet productivity between individual imMKCLs from a subclone with increased platelet productivity (subclone 38; red line) and imMKCLs from the bulk population (CL17, green line). Time-course imaging was performed as before (Fig 1B) except that confocal imaging was carried out with as 4x lens (due to the lower spatial resolution and fluorescence signal sensitivity under these experimental conditions, the number of PLPs detected is proportional to but likely lower than the actual number of PLPs produced by each cell). While cells from the subclone produce more PLPs (p = 3.449E-13; Student’s t-test), they still include cells the produce few if any PLPs. Representative screenshots (frame # 267) from the bulk and the subclone time-course datasets are shown next to the plot. A video with the time-course imaging data is included in the Supplemental Data section. (C) Stains/dyes used in the high-content chemical genetics screen (images from a spreading phenotype hit well). (D) Frequency distribution of compounds in the screening library. The screening library included several commercial compound libraries, resulting in some compounds being represented multiple times. (E) Image representing processing and analysis steps used to classify pixels as belonging to individual imMKCLs (including proplatelet extensions): background-corrected, cropped, and pseudo-colored RGB image representing the central region of a vinca alkaloid hit well (left), cell mask created to capture imMKCLs and their pro-platelet-like extensions but excluding debris and detached platelet-like particles (middle), and cells after removal of the debris/platelet-like particles and empty areas (right). Scale bar = 100μm. (F) Fiji and Python scripts were used to process, analyze, and plot the high-content high-thr [file pone.0326165.s001.tiff]

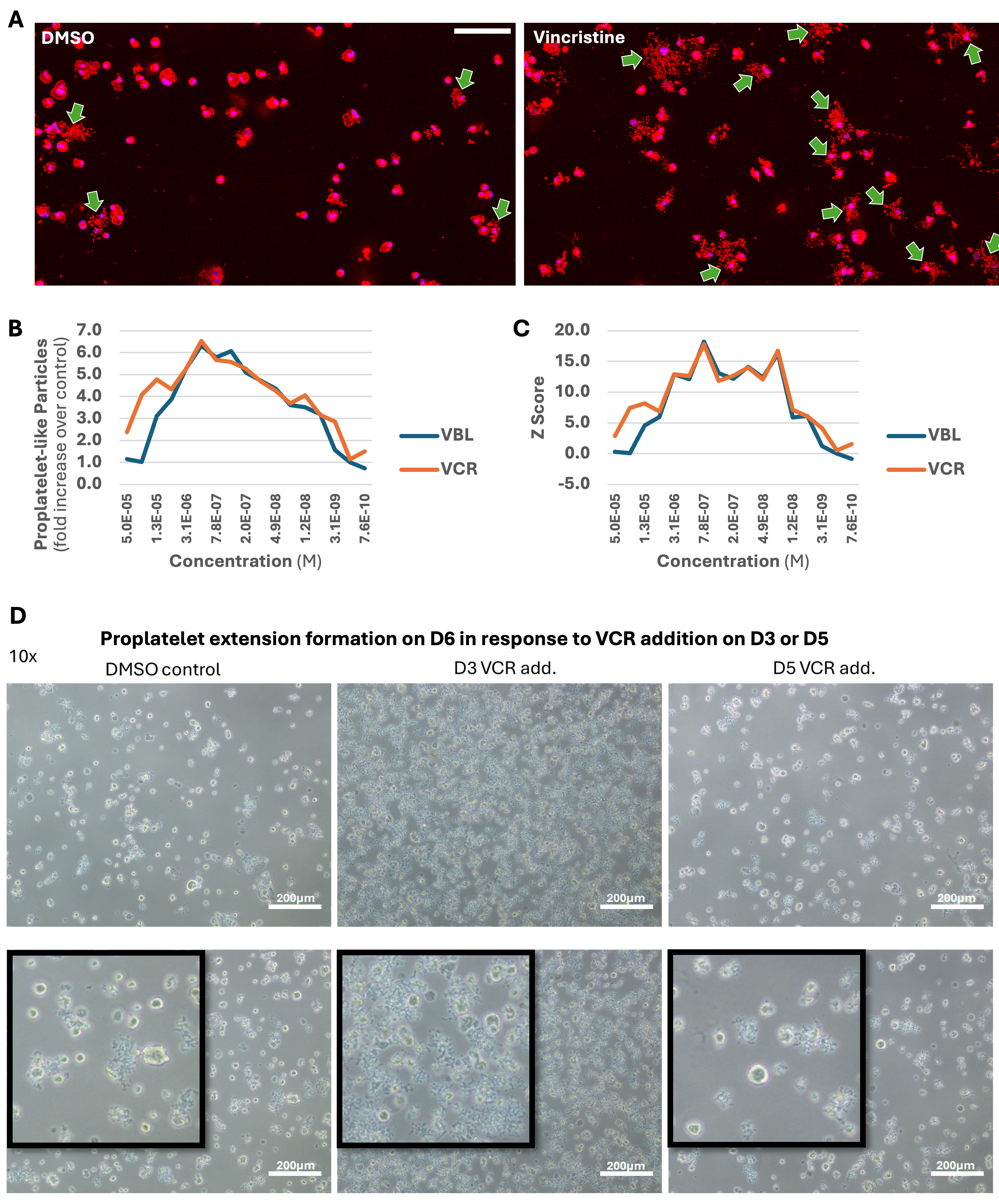

Supplement: S2 Fig — (A) Representative images (20x confocal) showing PKH426/Hoechst stained imMKCLs (D6) after treatment with DMSO or hit compound (VCR) under static conditions. Arrows mark imMKCLs with proplatelet extensions. (B) Titration of hit compounds vincristine (VCR) and vinblastine (VBL) under static culture conditions (384w plate). The fold-change (over control) of the number of platelet-like particles per cell is plotted over compound concentration. Processing and quantification of the 20x confocal images were carried out using a FIJI script. (C) Z-score plots of the data shown in (B). With both compounds, Z-scores of ≥6 were obtained with concentrations as low as 6.1nM. (D) Representative images (10x brightfield) of day 6 proplatelet formation in static imMKCL differentiation culture. 10nM VCR was added on day 3 or day 5. Scale bars represent 200µm. Black-framed views of 2x zoomed-in sections are included for better visibility. (TIFF) [file pone.0326165.s002.tiff]

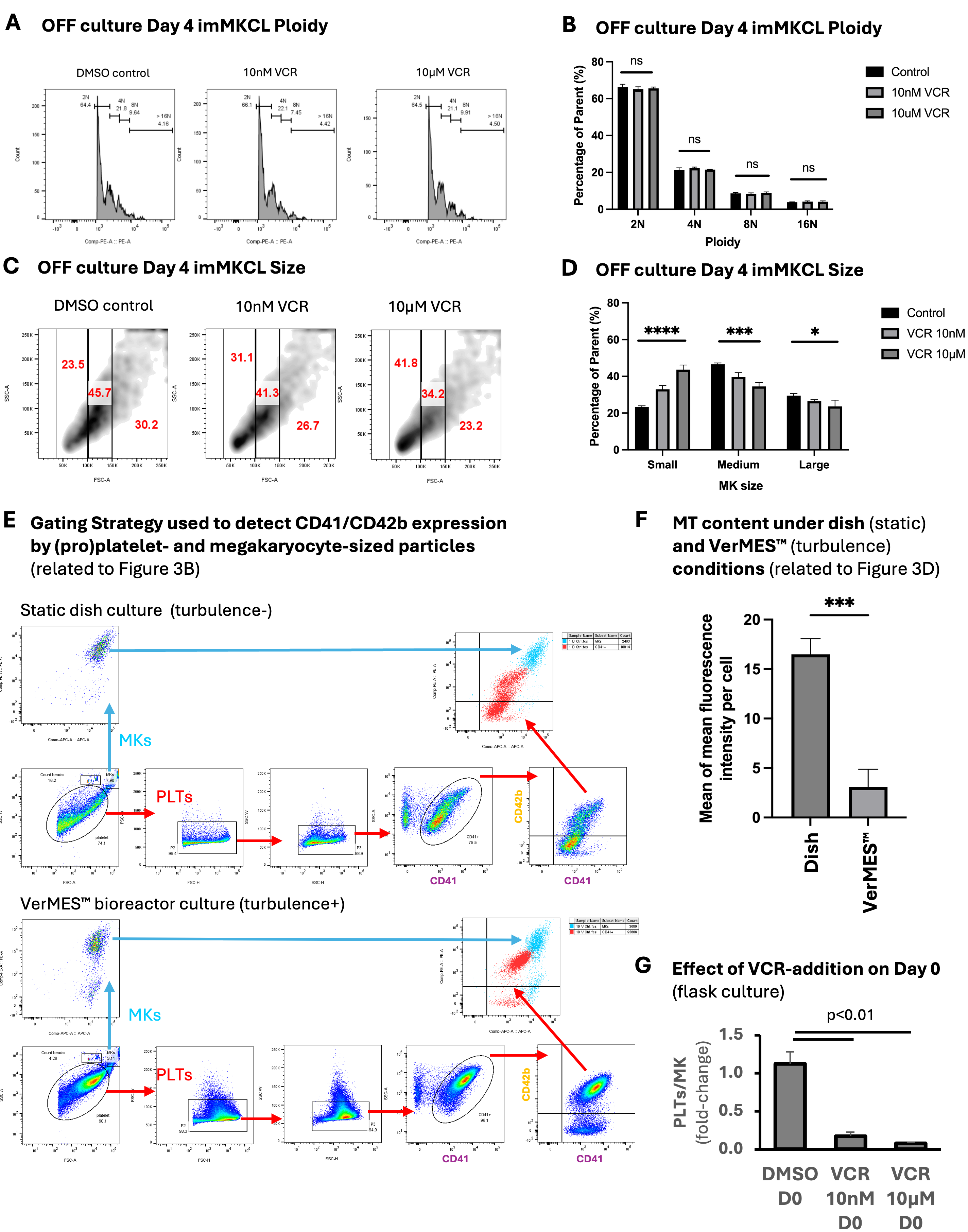

Supplement: S3 Fig — (A) Representative flowcytometry histograms showing day 4 maturing imMKCL ploidy under orbital shaking flask culture condition. DMSO, 10nM VCR, or 10µM VCR was added on day 3. Gates show 2N, 4N, 8N, > 16N. (B) Bar plot shows compiled data of day 4 maturing imMKCL under orbital shaking flask culture condition. DMSO, 10nM VCR, or 10µM VCR was added on day 3. Experiments were conducted n = 3 times. Error bars represent SEM and statistical analysis was conducted using one way-Anova test of variance. (C) Representative flow cytometry density plots for imMKCL cell size on day 4 of differentiation under orbital shaking flask culture condition (FSC-A). Gates show < 100K (left, small), 100K-150K (middle, medium), > 150K (right, large). Either DMSO, 10nM VCR, or 10µM VCR were added on day 3. (D) (E) Bar plot shows compiled data of day 4 differentiating imMKCLs under orbital shaking flask culture condition. DMSO, 10nM VCR, or 10µM VCR were added on day 3. Experiments were conducted n = 3. Error bars represent SEM and statistical analysis was conducted using one way-Anova test of variance. *p < 0.05, *** p < 0.001, ****p < 0.0001. Gating Strategy and Overlay of PLTs (pro)platelets) and MKs (imMKCLs) sampled on day 6 from dish (turbulence–) and VerMES™ (turbulence+) differentiation cultures (related to Fig 3B, C). (F) FIJI/ImageJ-based quantification of confocal imaging signal representing the MT content of imMKCLs sampled from day 4 static-dish and VerMES™ differentiation cultures (related to Fig 3D; Student’s t-Test, ***p < 0.001). (G) Effect on platelet-yields of early (day 0) addition of VCR to imMKCLs differentiating in shaker flasks. Platelet yields are graphed as fold-change in yield (PLTs/MK) over control (D0, DMSO). Data shows means (n = 3; error bars = σ; p-values: t-test). (TIFF) [file pone.0326165.s003.tiff]

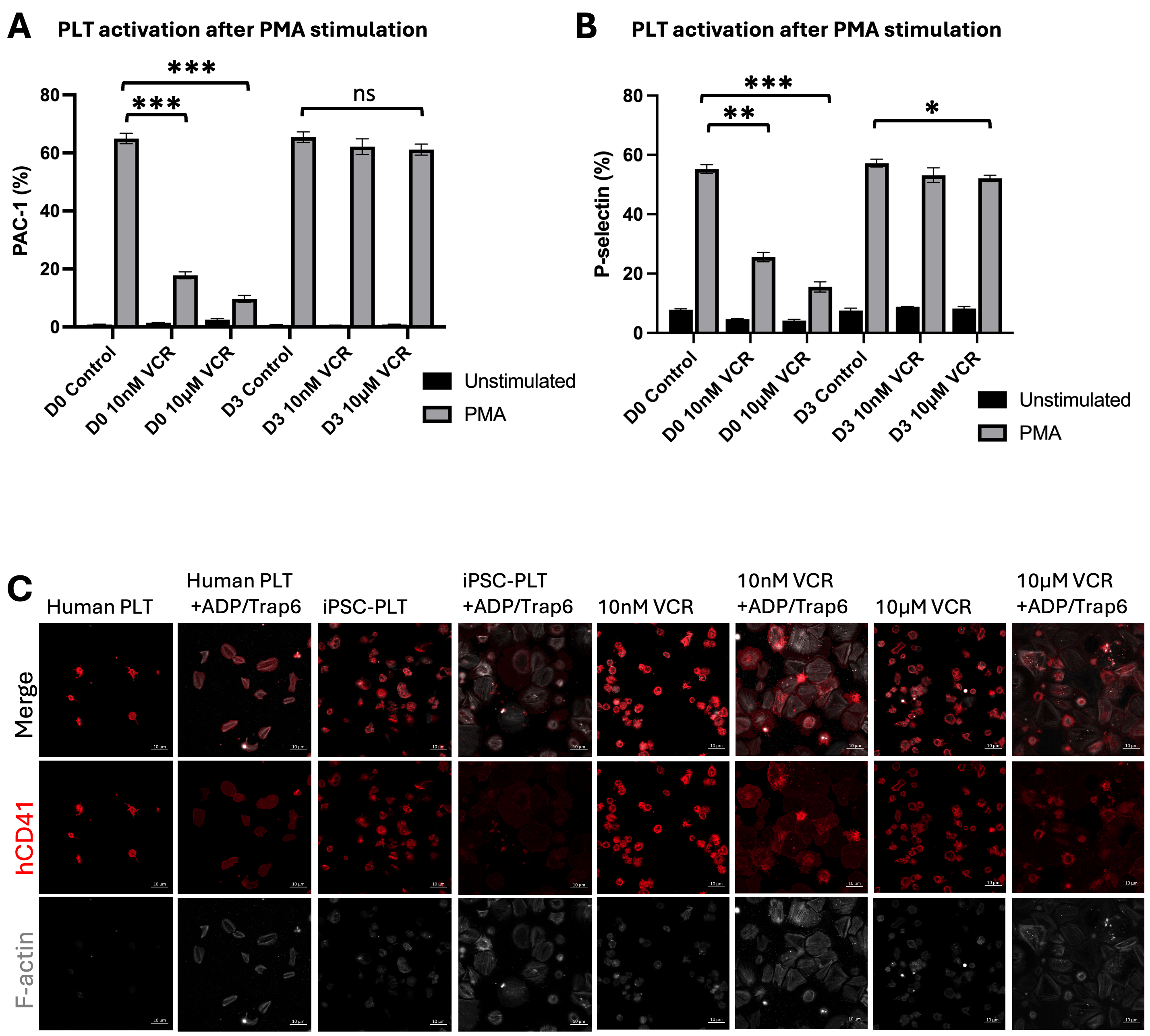

Supplement: S4 Fig — (A) Compiled data of flow cytometric analysis of PAC-1 epitope surface staining of unstimulated and PMA stimulated platelets harvested from imMKCLs in shaker-flask cultures, with and without imMKCL exposure to VCR at the indicated doses and times. Data shows means (n = 3; error bars = SEM; t-test: ns p > 0.05; *** p < 0.0001). (B) Compiled data of flow cytometric analysis of CD62P/P-Selectin epitope surface staining of unstimulated and ADP/TRAP-6 stimulated platelets harvested from imMKCLs in shaker-flask cultures, with and without imMKCL exposure to VCR at the indicated doses and times. Data shows means (n = 3; error bars = SEM; t-test: * p < 0.05; ** p < 0.001; *** p < 0.0001). (C) Confocal micrographs of platelet spreading. Human PLTs, iPSC-PLTs, 10nM VCR iPSC-PLTs, or 10µM VCR iPSC-PLTs were seeded on human fibrinogen coated dishes with or without ADP/Trap6. Red: hCD41 (anti-hCD41-APC); white: filamentous actin (SPY-555). Scale bar: 10µm. (TIFF) [file pone.0326165.s004.tiff]

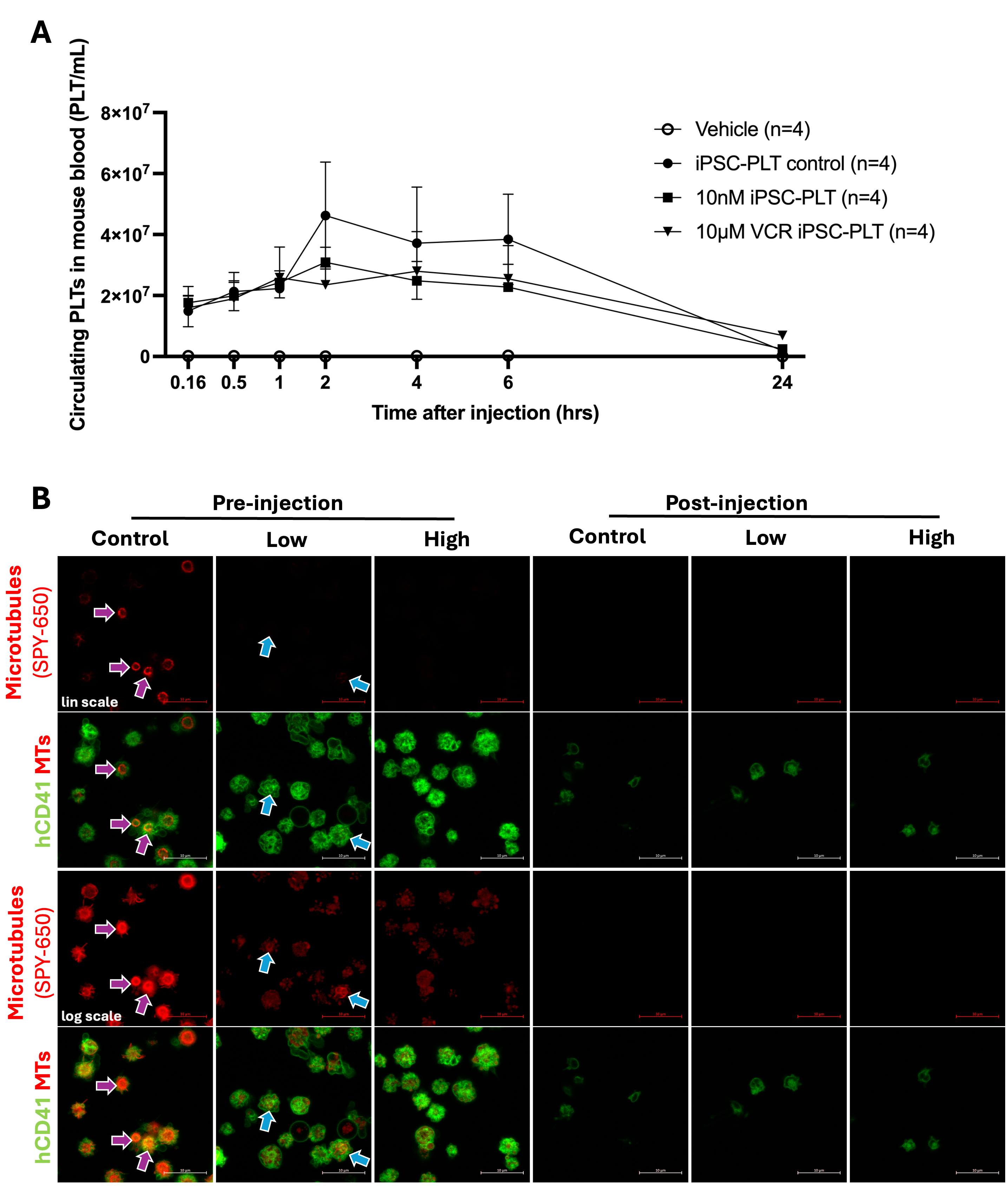

Supplement: S5 Fig — (A) iPSC-PLT circulation in mouse blood at time points: 10min, 30min, 1hr, 2hrs, 4hrs, 6hrs, 24hrs. n = 3 mice per group. Error bars represent SD. Related to Fig 5E. (B) Confocal micrographs of iPSC-PLTs from in vivo circulation experiment (performed in NSGS-SGM3 mice). Pre-injection(left) and post injection (right). Staining shows microtubules with SPY650-Tubulin (red) and with anti-hCD41 antibody (green). Scale bars show 10µm. Purple arrows point to prominent marginal band MTs present in control iPSC-PLTs. The tubulin staining signal is also shown in log-scale (lower half) to better visualize presence of residual MT staining and marginal band structures in 10 nM VCR iPSC-PLTs prior to injection (blue arrows). (TIFF) [file pone.0326165.s005.tiff]
