# Supplementary material for: Association of microtubule destabilization with platelet yields in terminally differentiating hiPSC-derived megakaryocyte lines
Source: PLoS One. 2025 Jun 25;20(6):e0326165. doi: 10.1371/journal.pone.0326165 (PMC12194186; doi:10.1371/journal.pone.0326165)
Supplement: S1 Table — Summary of the compound libraries used in the high throughput screen. (PDF) [file pone.0326165.s006.pdf]

S1 Table: List of Compound libraries

1536 well plates #	Libraries	No. of compounds
Plate 9, 19	Control	
Plates 1-6	Sigma LOPAC library (Sigma, LO1280) and 160 selected compounds	1440
Plates 7-8	ICCB known bioactive compound library (Enzo, BML-2840)	480
Plate 10-14	Selected Chembridge KINACore and Nuclear Hormone Receptor Core compounds	1200
Plate 15-17	Evotec NIH Clinical Collections 1+2 FDA approved drugs	720
Plate 18	Epigenetics Screening Library (Cayman, 11076) and 96 selected compounds	240
Libraries at ~10mM, compounds tested at 4 dilutions [1:500, 1:2,500, 1:12,500, 1:62,500]		
