# Supplementary material for: Association of microtubule destabilization with platelet yields in terminally differentiating hiPSC-derived megakaryocyte lines
Source: PLoS One. 2025 Jun 25;20(6):e0326165. doi: 10.1371/journal.pone.0326165 (PMC12194186; doi:10.1371/journal.pone.0326165)
Supplement: S2 Table — Vendor and catalog number or compound ID code for staining reagents, antibodies, and small molecules and other chemicals. (PDF) [file pone.0326165.s007.pdf]

S2 Table: List of Reagents

Reagent	CAT#, CAS ID, or InChIKey	Supplier
Vincristine	57-22-7	TOCRIS
Vincristine Sulfate	226-02303	Fujifilm Wako
Vinblastine	865-21-4	TOCRIS
AMG9810	545395-94-6	TOCRIS
Compound 4	BFEUNBCHKFWKFV-UHFFFAOYSA-N	Mcule
Compound 2	FZMKNCSSXNTEEI-UHFFFAOYSA-N	Mcule
Compound 3	UTPBOGSRVYQCPU-UHFFFAOYSA-N	Chembridge
Compound 1	FGTFVGBFMUEHQF-UHFFFAOYSA-N	Mcule
KP457	1365803-52-6	Medchem Express
Y-39983	199433-58-4	Medchem Express
GNF351	182707	Calbiochem
CD41a-PE	555467	BD Biosciences
CD41a- APC	303710	Biolegend
CD42b-APC	551061	BD Biosciences
CD42b-PE	303906	Biolegend
CD62p-PE	555524	BD Pharmingen™
CD62p-BV421	304926	Biolegend
PAC1-FITC	340507	BD Biosciences
Annexin V-FITC	556419	BD Biosciences
mCD41-PE-Cy7	133916	Biolegend
Plasmem Bright Green	P504	Dojindo Laboratories
SPY-555-FastAct	CY-SC202	Cytoskeleton Inc.
SPY-650-Tubulin	CY-SC503	Cytoskeleton Inc.
DAPI	D9542-1MG	Sigma-Aldrich
HOECHST-33342	H3570 RFP-50-5	Thermofisher
HOECHST-33342	62249	Thermofisher
Sytox Green	R37168	Thermofisher
PKH26	PKH26GL-1KT T5168-100UL	Sigma-Aldrich
Ionomycin	091-05833	Wako
ADP	A-2754	Sigma Aldrich
Trap-6	H-8365.0005	BACHEM
EDTA	06894-14	Nacalai Tesque
PMA	P1585-1MG	Sigma Aldrich
MitoBright LT Green	MT10	Dojindo Laboratories
